# Early Prediction of High-Flow Oxygen Therapy Failure in COVID-19 Acute Hypoxemic Respiratory Failure: A Retrospective Study of Scores and Thresholds

**DOI:** 10.7759/cureus.32087

**Published:** 2022-11-30

**Authors:** Mircea T Talpoș, Anaximandre Rasson, Christophe De Terwangne, Olivier Simonet, Fabio S Taccone, Frédéric Vallot

**Affiliations:** 1 Intensive Care Unit, Centre Hospitalier de Wallonie picarde, Tournai, BEL; 2 Geriatrics, Université catholique de Louvain, Brussels, BEL; 3 Intensive Care Unit, Hôpital Erasme, Université libre de Bruxelles, Brussels, BEL

**Keywords:** modified rox index, patient self-induced lung injury, ards (acute respiratory distress syndrome), invasive mechanical ventilation, non-invasive ventilaton, sars-cov-2, covid 19, rox index, acute hypoxemic respiratory failure, high flow oxygenation

## Abstract

Background

High-flow oxygen therapy (HFOT) has been widely used as an effective alternative to invasive mechanical ventilation (IMV) in some critically ill patients with COVID-19 pneumonia. This study aimed to compare different tools, including the respiratory rate and oxygenation (ROX) index, to predict HFOT failure in this setting.

Methodology

This single-center retrospective observational study was conducted from September to December 2020 and assessed COVID-19 patients who required HFOT as the first treatment at admission; HFOT failure was defined as IMV use. Prognostic scoring tools were as follows: the Sequential Organ Failure Assessment (SOFA), Acute Physiology And Chronic Health Evaluation (APACHE) II, and Simplified Acute Physiology Score (SAPS) III scores; C-reactive protein; lung consolidation percentage on chest CT; mean partial pressure of oxygen in arterial blood (PaO_2_)/fraction of inspired oxygen (FiO_2_) ratio; and ROX index and modified ROX index, calculated using PaO_2_ instead of blood oxygen saturation, within the first 24 hours after admission to the intensive care unit (ICU). These scores were analyzed using a multivariate Cox proportional hazard model; optimal cutoffs were computed using the R system for statistical computing.

Results

The study enrolled 52 patients, 31 (60%) of whom experienced HFOT failure. The best predictors of HFOT failure measured 24 hours after HFOT initiation were as follows: PaO_2_/FiO_2_ (threshold 123.6, sensitivity 87%, specificity 81%, hazard ratio [HR] 7.76, and 95% confidence interval [CI] 2.39-17.1); ROX index (threshold 5.63, sensitivity 68%, specificity 95%, HR 6.18, and 95% CI 2.54-13.4); and modified ROX index (threshold 4.94, sensitivity 81%, specificity 90%, HR 8.16, and 95% CI 3.16-21.5) (*P* < 0.001 for all).

Conclusions

Early assessment of the ROX index, modified ROX index, and PaO_2_/FiO_2_ ratio can adequately predict, with high accuracy, HFOT failure in COVID-19 patients. Because thresholds remain debated and are still not sufficiently validated, we advocate using them with caution for clinical decision-making in this context.

## Introduction

The initial management of severe acute hypoxemic respiratory failure (AHRF) caused by COVID-19 was based on the use of early invasive mechanical ventilation (IMV) [1]. An understanding of respiratory mechanics and severe hypoxemia in this setting has been of great importance in optimizing treatment for the routine implementation of non-invasive respiratory support. Therefore, conventional oxygen therapy, high-flow oxygen therapy (HFOT), continuous positive airway pressure (CPAP), and awake prone positioning have been evaluated as first-line treatments to reverse hypoxemia, reduce patient discomfort, and avoid the need for IMV [2-4]. Randomized clinical trials and prospective studies suggest early administration of HFOT or CPAP instead of conventional oxygen therapy in AHRF to avoid IMV [5-7]; bioaerosols generated by these maneuvers are considered negligible, according to available evidence [8].

The pathophysiology of COVID-19-related AHRF involves a complex immune response with activation of cytokines, diffuse lung inflammation, and endothelial damage. The consequences of heterogeneous respiratory mechanics in this setting imply severe alveolar and interstitial edema with increased shunt, microthrombosis, loss of pulmonary perfusion, and progression to acute respiratory distress syndrome [9-11]. If the respiratory rate (RR), gas exchange, and spontaneous tidal volume are not improved under HFOT or CPAP, the patient’s respiratory drive is suggested to induce a patient self-inflicted lung injury (P-SILI) in close relation to refractory hypoxemia, edema, and atelectasis. Thus, early identification of patients at risk of HFOT failure and the timing of IMV for better outcomes still are of major interest.

Among various predictors of respiratory failure caused by bacterial or viral pneumonia, the partial pressure of oxygen in arterial blood (PaO_2_)/fraction of inspired oxygen (FiO_2_) ratio, and the RR and oxygenation (ROX) index have been used as early tools to identify non-responders to HFOT, with variations in reported cut-offs for failure and risk of endotracheal intubation [12-17]. Two systematic reviews [13,18] have concluded a good predictive value of the ROX index in COVID-19 AHRF, in which studies took measurements during 2-12 hours after the onset of therapy. The threshold values for the ROX index to predict failure ranged from 2.7 to 5.9 (95% confidence interval [CI] 4.2-5.4).

Our study aimed to address and retrospectively compare different scores at admission and up to 24 hours that can accurately predict HFOT failure in AHRF secondary to COVID-19. It has the novelty of evaluating the prognostic potential of the mean values of the ROX index and modified ROX index.

## Materials and methods

Study design and population

We conducted a retrospective study in a general hospital in Wallonia, Belgium. We reviewed the hospital’s single-center electronic observational database (Xperthis Electronic Patient Record [EPR], Brussels, Belgium) and enrolled patients aged >18 years who were admitted to the intensive care unit (ICU) between September 1 and December 31, 2020, with the diagnosis of AHRF or acute respiratory distress syndrome [19], a positive real-time polymerase chain reaction (RT-PCR) assay for SARS-COV-2 and treated with HFOT in the first 24 hours after admission to the ICU. All patients tested positive for the SARS-COV-2 variant of concern (VOC) 202012/01, known as the Alpha variant (B.1.1.7). Patients were excluded if they had a defined therapeutic limitation (i.e., not to be resuscitated) or if they had HFOT, CPAP, or IMV before admission to the ICU.

Patients were classified into one of two groups based on their clinical response to HFOT: nonresponders, for whom HFOT failed and IMV support was required after their ICU admission, and responders, for whom success with HFOT was demonstrated by clinical improvement. The study was carried out according to the Declaration of Helsinki [20] concerning human experiments and followed the recommendations of *Good Clinical Practice* for information security and patient confidentiality [21]. The Local Ethical Committee approved the retrospective study protocol (reference number P202 2021) and waived the need for informed consent.

Data collection

We collected demographic data; chronic diseases and treatment; blood test results on admission; vital signs on admission; the estimated percentage of ground glass findings on chest CT, assessed by one radiologist through a nonstandardized method; and length of stay in the ICU. The Sequential Organ Failure Assessment (SOFA) score, the Acute Physiology and Chronic Health Evaluation (APACHE) II score, and the Simplified Acute Physiology Score (SAPS) III were calculated retrospectively after the first 24 hours of ICU admission. For each patient, a mean value was calculated for the ROX index, modified ROX index, and PaO_2_/FiO_2_ ratio using six measurements taken during the first 24 hours in the ICU. The ROX index was calculated as follows: blood oxygen saturation (SpO_2_/FiO_2_)/RR; the modified ROX index was calculated using PaO_2_ instead of SpO_2_: (PaO_2_/FiO_2_)/RR [22]. Two investigators (MTT and AR) independently reviewed patients for inclusion from the electronic database, AR evaluated the time to HFOT failure, and any disagreement was discussed with a third investigator (FV).

Patient management

Patients were admitted to the ICU from the emergency department or the ward with signs of AHRF, with an early warning score of 5 or higher, or if the following values were met: SpO_2_ < 90%, RR > 25/minute, PaO_2_ < 70 mmHg under 15 L/minute of oxygen under face mask, PaO_2_/FiO_2_ ratio < 200 mmHg, and chest CT with moderate-to-severe lung infiltration (i.e., >50% of lung volume). HFOT and CPAP were not administered before ICU hospitalization. At the time of the initiation of HFOT (OptiFlow/Airvo2 Fisher & Paykel Healthcare, Auckland, New Zealand, or Elisa 800 Löwenstein Medical, Hamburg, Germany), the SpO_2_, PaO_2_/FiO_2_ ratio, RR, and vital signs were encoded by a nurse. HFOT values were established at first at 1.0 FiO_2_ and airflow at 60 L/minute, adapted accordingly to reach each patient’s SpO_2_ > 92% and arterial PaO_2_ > 70 mmHg.

Endotracheal intubation and IMV of patients were considered in the following settings: persistent hypoxemia with PaO_2_ < 70 mmHg, PaO_2_/FiO_2_ ratio < 100 mmHg, or SpO_2_ < 90% at 1.0 FiO_2_; respiratory distress (RR > 25/minute, dyspnea with the use of accessory respiratory muscles, paradoxical breathing) and/or sweating; and increase in lactate levels from baseline and alteration of consciousness with a Glasgow Coma Scale score less than 9.

Every patient received 10 mg of intravenous dexamethasone at the start of HFOT for a total of 10 days. The clinical agitation or anxiety of each patient was assessed using the Richmond Agitation Sedation Scale (RASS) and adapted to an RASS score of 0 by using intravenous dexmedetomidine, starting with an initial infusion rate of 0.7 microg/kg/hour, adjusted stepwise within the dose range 0.2-1.4 microg/kg/hour. All patients had continuous monitoring of vital signs and arterial and central vein catheterizations. No patient had an awake prone positioning session at the time of the study.

Statistical analysis

Descriptive statistics were computed for all study variables. Discrete variables were expressed as percentages and continuous variables as mean (standard deviation) or median (25th-75th percentiles) as appropriate. Differences between HFOT success and failure were assessed using the chi-square test for categorical variables and the Kruskal-Wallis test for continuous variables. Receiver operating characteristic curves were computed to analyze the performance of potential predictors to correctly detect the independent variable (HFOT failure). The area under the curve (AUC) and appropriate metrics (sensitivity and specificity) were reported. Differences between AUC were analyzed using the DeLong method [23]. The maximum sum of sensitivity and specificity was used to estimate the optimal thresholds of the continuous variables of interest. The time to IMV using these cutoffs was presented as a Kaplan-Meier curve. The curves were compared with the log-rank test to assess the difference in the probability of HFOT failure at any time during therapy to distinguish between predictive failure and predictive success.

Multivariate Cox proportional hazard models were performed to analyze the risk of HFOT failure during therapy using the PaO_2_/RR ratio, the ROX index, and the modified ROX index. Different models were computed and adjusted for potential confounders. Variables showing collinearity (i.e., a variance inflation factor > 5) were excluded before modeling; only variables associated with HFOT failure in the univariate analysis (*P*-value < 0.2) were included in the multivariate model. Three models, each with one predictor studied (PaO_2_/FiO_2_ ratio, ROX index, and modified ROX index), were retained for comparison.

Throughout the analysis, *P* < 0.05 was considered statistically significant. Statistical analysis was performed using R: A Language and Environment for Statistical Computing (R Core Team, Vienna, Austria, 2020, https://www.r-project.org/ [used packages car, caret, gtsummary, pROC, cutpointr, ggplot, survival, forestmodel]).

## Results

Study population

**Figure 1:**
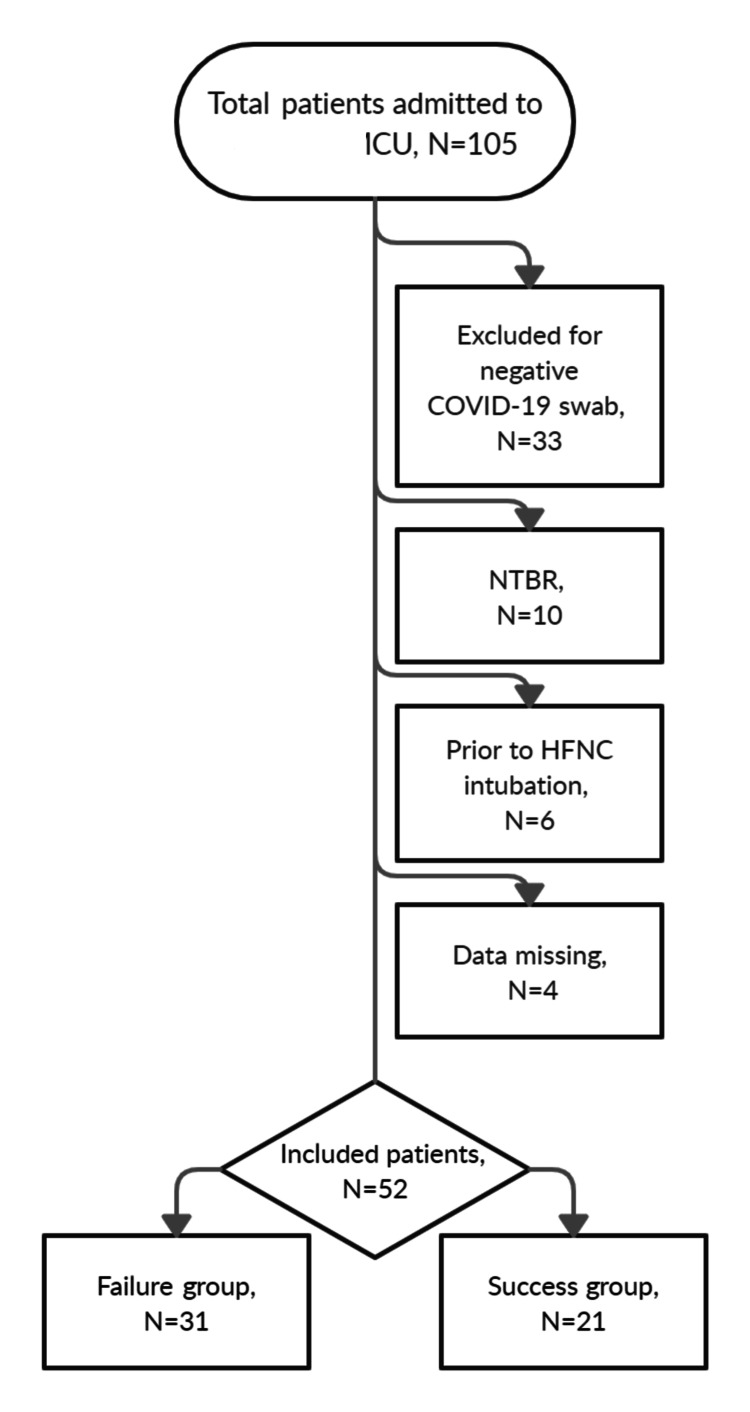
Flowchart of the cohort. Figure credits:  Mircea T. Talpoș ICU, intensive care unit; N, number of patients

During the study period, a total of 105 patients with AHRF were admitted to the ICU; 33 patients with negative RT-PCR assays for COVID-19 were excluded. Of the remaining 72 patients, 10 made a decision to limit life-sustaining therapy, 6 were intubated before admission to the ICU, and 4 had missing data on respiratory values and blood gas analysis at admission, resulting in 52 patients for the final analysis (Figure 1).

The baseline of patient characteristics on admission is presented in Tables 1, 2. A total of 31 (60%) patients were included in the HFOT failure group; they were intubated secondary to persistent acute respiratory failure under HFOT. Of these 31 patients, mortality at 28 days was 68% (21 patients). The main causes of death were refractory hypoxemia (11 patients), multiple organ failure (7 patients), cardiac arrest (2 patients), and cardiogenic shock (1 patient). Additionally, in the HFOT success group, 1 (5%) patient died after being weaned from HFOT.

**Table 1:** Population characteristics: comorbidities and vital signs at admission according to HFOT failure. HFOT, high-flow oxygen therapy; *N*, total patients included; *n*, number of patients in the category; BMI, body mass index; HTA, arterial hypertension; COPD, chronic obstructive pulmonary disease; CHADSVASC, congestive heart failure, hypertension, age ≥ 75 years, diabetes, stroke, vascular disease, age 65 to 74 years, and sex category (female) score for atrial fibrillation stroke risk

Characteristics	Mean and range (*N* = 52)	HFOT		*P*-value
		Success (*n* = 21)	Failure (*n* = 31)	
Demographics				
Male gender (*n*)	35 (67%)	13 (62%)	22 (71%)	0.70
Age (years)	70 (65, 74)	68 (64, 73)	71 (65, 76)	0.49
BMI (kg/m^2^)	30 (26, 36)	28 (25, 33)	31 (27, 37)	0.20
Comorbidities				
Chronic kidney failure (*n*)	9 (17%)	4 (19%)	5 (16%)	0.82
Cancer (*n*)	6 (11%)	1 (4.7%)	5 (16%)	0.38
HTA (*n*)	38 (73%)	15 (71%)	23 (74%)	>0.99
Hypercholesterolemia (*n*)	19 (36%)	6 (28%)	13 (42%)	0.67
Chronic atrial fibrillation (*n*)	13 (26%)	3 (14%)	10 (32%)	0.34
Ischemic heart disease (*n*)	4 (8.0%)	1 (4.7%)	3 (9.7%)	>0.99
Peripheral artery disease (*n*)	4 (8.0%)	1 (4.7%)	3 (9.7%)	>0.99
Stroke	3 (6.0%)	0 (0%)	3 (9.7%)	0.28
COPD	19 (37%)	5 (24%)	14 (45%)	0.30
Diabetes mellitus	22 (42%)	8 (38%)	14 (45%)	>0.99
Rheumatic disease	3 (6.0%)	1 (4.7%)	2 (6.5%)	>0.99
Charlson comorbidity index (points)	4.00 (3.00, 6.00)	4.00 (2.50, 6.00)	5.00 (3.00, 6.50)	0.29
CHADSVASC score	3.00 (2.00, 3.00)	2.00 (2.00, 3.50)	3.00 (2.00, 3.00)	0.40
Admission vitals				
Systolic blood pressure (mmHg)	132 (119, 144)	132 (117, 146)	130 (119, 142)	0.79
Diastolic blood pressure (mmHg)	68 (59, 80)	71 (60, 87)	68 (58, 75)	0.29
Cardiac rate (beats per minute)	89 (77, 106)	86 (78, 107)	92 (78, 104)	0.75
Respiratory rate (breaths per minute)	28.0 (23.2, 31.0)	27.0 (23.0, 30.5)	28.0 (23.5, 31.0)	0.76
Body temperature (°C)	36.90 (36.50, 37.70)	36.70 (36.45, 37.15)	37.20 (36.55, 38.35)	0.11
Pulse oximetry (%)	87.5 (82.0, 90.8)	89.0 (84.5, 90.5)	87.0 (80.5, 90.5)	0.69

**Table 2:** Population characteristics: admission biology and severity scores according to HFOT failure. *N*, total patients included; *n*, number of patients in the category; HFOT, high-flow oxygen therapy; N/L, neutrophil-to-lymphocyte ratio; PLT, platelets count; PLR or P/L ratio, platelet-to-lymphocyte ratio; SII, systemic immune inflammation index; CRP, C reactive protein; chest CT, chest computed tomography; SOFA, Sequential Organ Failure Assessment; APACHE, Acute Physiology and Chronic Health Evaluation; SAPS, Simplified Acute Physiology Score; COT, conventional oxygen therapy; RR, respiratory rate; FiO_2_, fraction of inspired oxygen; SpO_2_, pulse oximetry; PaO_2_, partial pressure of oxygen in arterial blood

Characteristics	Mean and range (*N* = 52)	HFOT		*P*-value
		Success (*n* = 21)	Failure (*n* = 31)	
Admission biology				
White blood cell count/mm^3^	9,730 (7,345, 13,532)	9,510 (7,535, 11,280)	9,950 (7,305, 14,170)	0.94
Neutrophils (mm^3^)	8,379 (5,730, 11,228)	8,282 (5,984, 10,060)	8,577 (5,668, 12,440)	0.58
Lymphocytes(mm^3^)	631 (382, 860)	757 (370, 1,102)	598 (382, 767)	0.41
N/L ratio	13 (7, 25)	11 (6, 18)	13 (8, 25)	0.45
PLT × 10^3^ (mm^3^)	230 (188, 280)	256 (184, 278)	214 (188, 280)	0.62
P/L ratio (PLR)	0.37 (0.22, 0.59)	0.44 (0.19, 0.61)	0.35 (0.25, 0.56)	0.94
SII (×10^9^ cells/L)	673 (51, 2,777)	777 (58, 2,808)	261 (38, 2,147)	0.79
CRP (mg/dL)	107 (77, 174)	91 (56, 114)	116 (92, 208)	0.027
Fibrinogen (mg/dL)	645 (532, 734)	623 (574, 720)	687 (532, 760)	0.31
Severity scores, first 24 hours				
Days before ICU admission	2.00 (0.00, 5.00)	1.00 (0.00, 4.00)	2.00 (0.00, 6.00)	0.37
Chest CT COVID-19 ground glass findings (%)	60 (50, 75)	50 (30, 60)	70 (50, 75)	0.009
SOFA score at 24 hours	2.00 (2.00, 4.00)	2.00 (2.00, 3.00)	3.00 (2.00, 4.00)	0.047
APACHE II	11.0 (8.0, 13.0)	9.0 (8.0, 12.0)	11.0 (9.2, 13.0)	0.13
SAPS III	3.0 (2.0, 6.0)	2.0 (1.0, 4.0)	3.5 (2.0, 6.0)	0.13
COT before HFOT (days)	1.00 (1.00, 2.00)	1.00 (1.00, 2.00)	1.00 (1.00, 2.00)	0.57
HFOT (days)	4.0 (3.0, 6.0)	5.0 (3.0, 6.0)	3.0 (2.0, 5.0)	0.060
Days till endpoint	5.0 (3.8, 8.0)	6.0 (5.0, 7.0)	5.0 (3.0, 8.0)	0.29
RR, mean first 24 hours	22.9 (20.3, 26.6)	22.1 (19.7, 23.3)	25.3 (20.9, 28.1)	0.037
FiO_2_, mean first 24 hours	0.74 (0.56, 0.88)	0.56 (0.52, 0.70)	0.88 (0.74, 0.90)	<0.001
SpO_2_, mean first 24 hours	92.8 (91.3, 94.1)	93.6 (92.7, 95.0)	92.4 (91.1, 93.3)	0.010
PaO_2_, mean first 24 hours	71 (67, 76)	73 (71, 83)	70 (64, 73)	0.004
PaO_2_/FiO_2_, mean first 24 hours	98 (80, 137)	137 (126, 147)	82 (73, 98)	<0.001
ROX index, mean first 24 hours	6.15 (4.49, 7.67)	7.71 (6.68, 9.08)	5.13 (4.06, 6.38)	<0.001
Modified ROX index, mean first 24 hours	4.89 (3.41, 6.23)	6.79 (5.43, 7.25)	4.08 (2.82, 4.82)	<0.001
Outcomes				
Dead	22 (42%)	1 (4.8%)	21 (68%)	<0.001

HFOT success versus failure

Analysis of population demographics showed that the HFOT failure group had significantly higher C-reactive protein (CRP) values and more lung infiltrates on chest CT than the success group at admission. At 24 hours after admission, a higher SOFA score, higher RR, and increased FiO_2_ supply were observed in the HFOT failure group, associated with lower PaO_2_, PaO_2_/FiO_2_ ratio, ROX index, and modified ROX index, compared with the HFOT success group.

Furthermore, no statistical differences were shown between the two groups regarding the Charlson comorbidity index, comorbidities, baseline laboratory tests (other than CRP), severity scores (APACHE II and SAPS III) at admission, and length of stay in the ICU.

**Figure 2:**
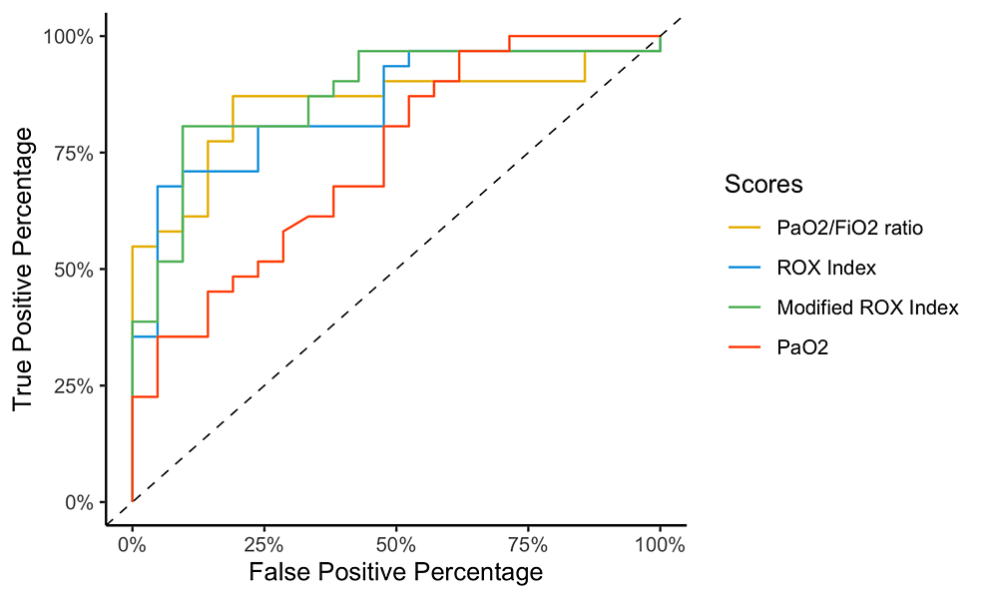
Receiver operating curve plots for the PaO2/FiO2 ratio, ROX index, modified ROX index, and PaO2 at 24 hours to predict the outcome of high-flow oxygen therapy. PaO_2_/FiO_2_, partial pressure of oxygen in arterial blood to the fraction of inspired oxygen ratio; ROX, respiratory rate and oxygenation index

After computing the AUC for the prediction capacity of HFOT failure, the modified ROX index showed the best value (0.87), followed by the PaO_2_/FiO_2_ ratio (0.85) and ROX index (0.85), although these values were not statistically different among them (Figure 2). The PaO_2_ measure showed the lowest AUC (0.74) to predict HFOT failure, significantly lower than that for the modified ROX index (*P* = 0.04).

**Figure 3:**
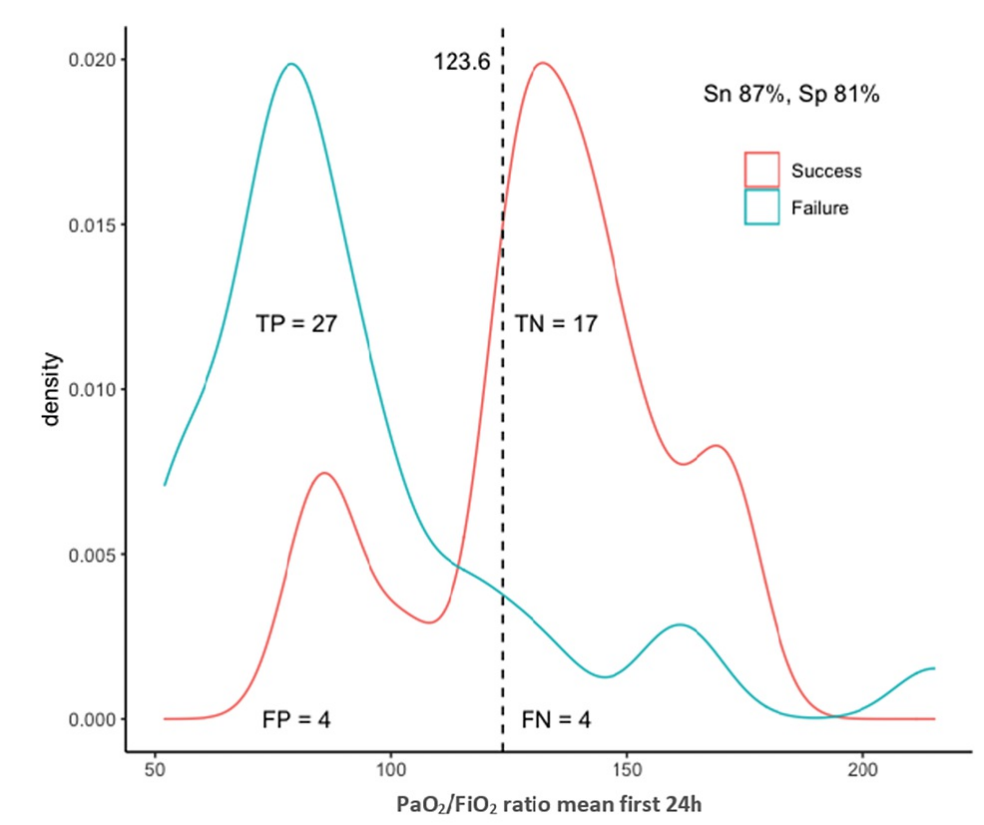
Optimal threshold analysis for the PaO2/FiO2 ratio TP, true positives; TN, true negatives; FP, false positives; FN, false negatives; Sn, sensitivity; Sp, specificity; PaO_2_/FiO_2_, partial pressure of oxygen in arterial blood to the fraction of inspired oxygen ratio

**Figure 4:**
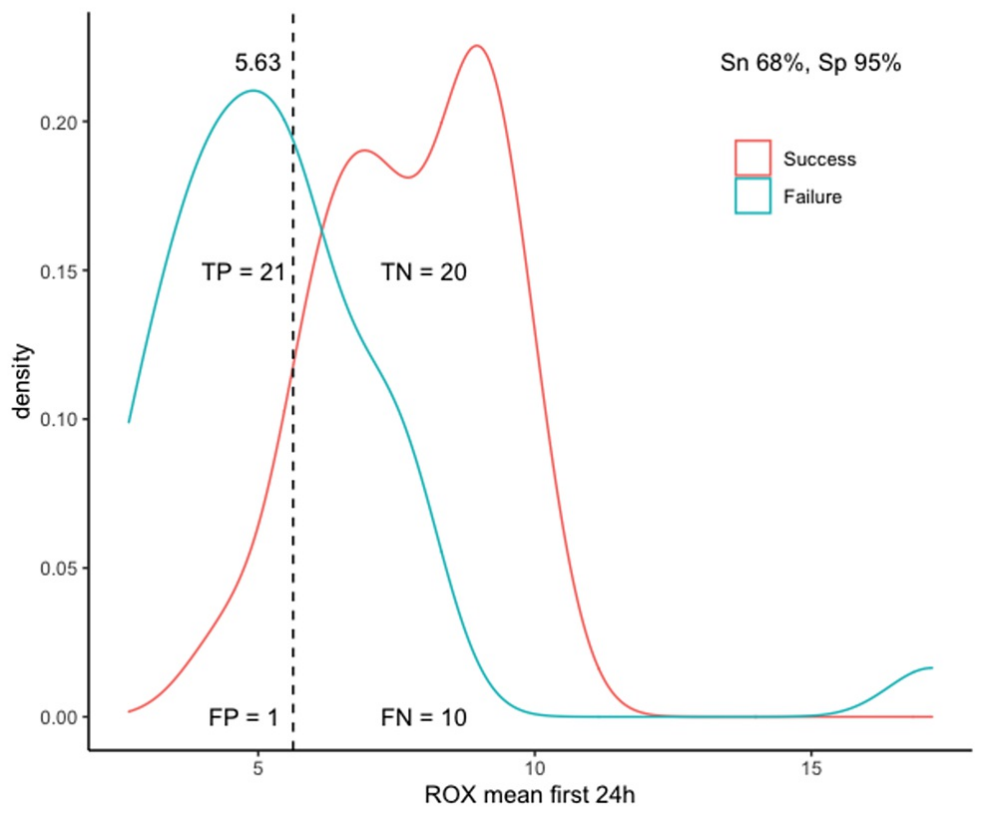
Optimal threshold analysis for the ROX index. TP, true positives; TN, true negatives; FP, false positives; FN, false negatives; Sn, sensitivity; Sp, specificity; ROX, respiratory rate and oxygenation index

**Figure 5:**
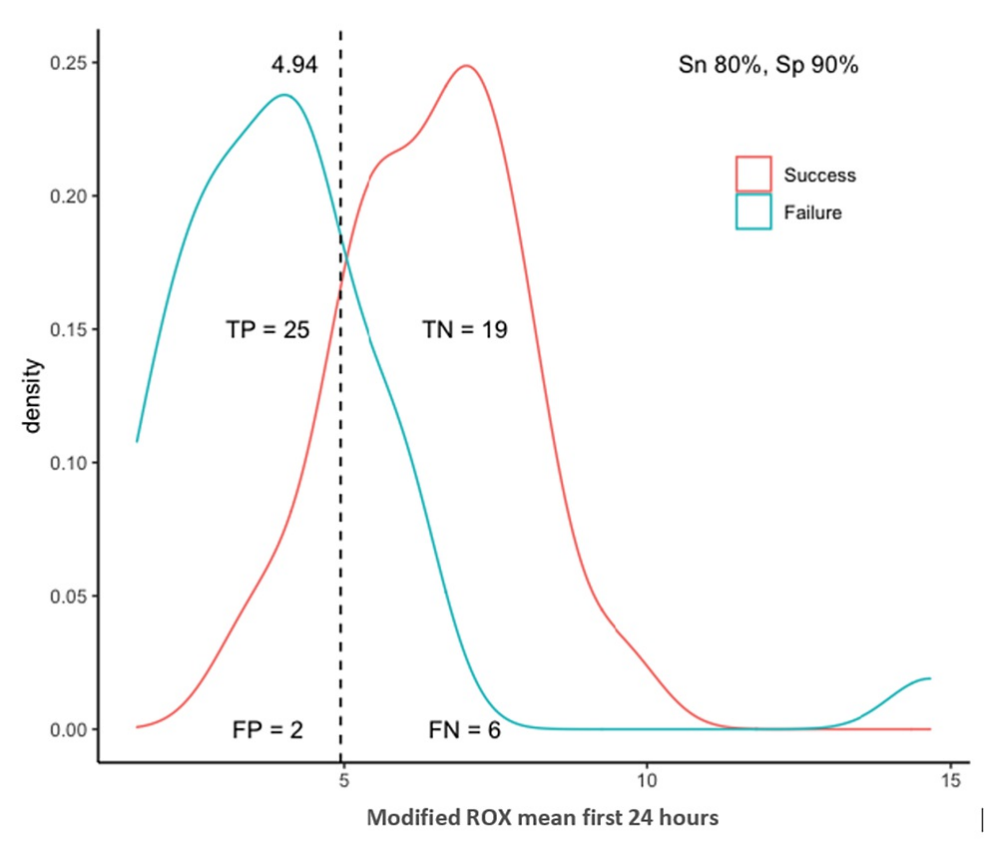
Optimal thresholds analysis for the modified ROX index. TP, true positives; TN, true negatives; FP, false positives; FN, false negatives; Sn, sensitivity; Sp, specificity; ROX, respiratory rate and oxygenation index

The optimal identified threshold for the PaO_2_/FiO_2_ ratio to predict failure of HFOT was 123.6, with a sensitivity of 87% and specificity of 81%; the optimal threshold for the ROX index was 5.63, with a sensitivity of 68% and specificity of 95%; and the optimal threshold for the modified ROX index was 4.94, with a sensitivity of 81% and specificity of 90% (Table 3, Figures 3-5).

**Table 3:** Metrics of optimal cut point analysis of PaO2/FiO2 ratio, ROX index, and modified ROX index. AUC, area under the curve; PaO_2_/FiO_2_, partial pressure of oxygen in arterial blood to the fraction of inspired oxygen ratio; ROX, respiratory rate and oxygenation index

Score	AUC	Optimal cut point	Accuracy	Sensitivity	Specificity
PaO_2_/FiO_2_ ratio	0.851	123.6	0.84	0.87	0.81
ROX index	0.848	5.63	0.79	0.68	0.95
Modified ROX index	0.872	4.94	0.85	0.81	0.90

To analyze the risk factors for HFOT failure over time, three multivariate Cox proportional hazard models were calculated. Using an adjustment for CRP values, lung involvement on chest CT, and the SOFA score, all three variables (PaO_2_/FiO_2_ ratio, hazard ratio [HR] 7.76 [95% CI 2.39-17.1]; ROX index, HR 6.18 [95% CI 2.54-13.4]; modified ROX index, HR 8.16 [95% CI 3.16-21.5]) were independently associated with an increased risk of failure of HFOT over time. All three models had significant overall goodness of fit (likelihood ratio [LR] test < 0.001). In particular, the model with the modified ROX index showed a slightly better performance compared to the others (Akaike information criterion [AIC] modified ROX index of 178, versus AIC PaO_2_/FiO_2_ ratio of 181 and AIC ROX index of 183), as shown in Table 4.

**Table 4:** . Multivariate COX proportional hazard modeling. HR, hazard ratio; CI, confidence interval; AIC, Akaike information criterion; LR, likelihood ratio test; PaO_2_/FiO_2_, partial pressure of oxygen in arterial blood to the fraction of inspired oxygen ratio; ROX, respiratory rate and oxygenation index; CRP, C-reactive protein; CT, computed tomography; SOFA, Sequential Organ Failure Assessment

	HR	95% CI	P-value	AIC	LR
Model 1: PaO_2_/FiO_2_ ratio				181	<0.001
PaO_2_/FiO_2_	6.40	2.39, 17.1	<0.001		
CRP at admission	1.01	1.00, 1.01	0.004		
COVID-19 extension on chest CT	1.03	1.01, 1.06	0.012		
SOFA score at 24 hours	1.05	0.79, 1.39	0.755		
Model 2: ROX index				183	<0.001
ROX index	5.83	2.54, 13.4	<0.001		
CRP at admission	1.00	1.00, 1.01	0.035		
COVID-19 extension on chest CT	1.03	1.00, 1.05	0.025		
SOFA score at 24 hours	1.07	0.83, 1.38	0.597		
Model 3: modified ROX index				178	<0.001
Modified ROX index	8.24	3.16, 21.5	<0.001		
CRP at admission	1.01	1.00, 1.01	0.007		
COVID-19 extension on chest CT	1.03	1.00, 1.05	0.027		
SOFA score at 24 hours	1.11	0.85, 1.46	0.444		

**Figure 6:**
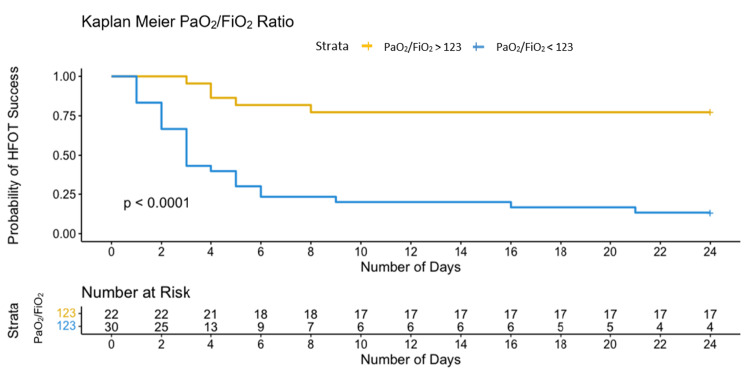
Kaplan-Meier curves of the probability of HFOT failure based on selected threshold for PaO2/FiO2. PaO_2_/FiO_2_, partial pressure of oxygen in arterial blood to the fraction of inspired oxygen ratio; HFOT, high-flow oxygen therapy

**Figure 7:**
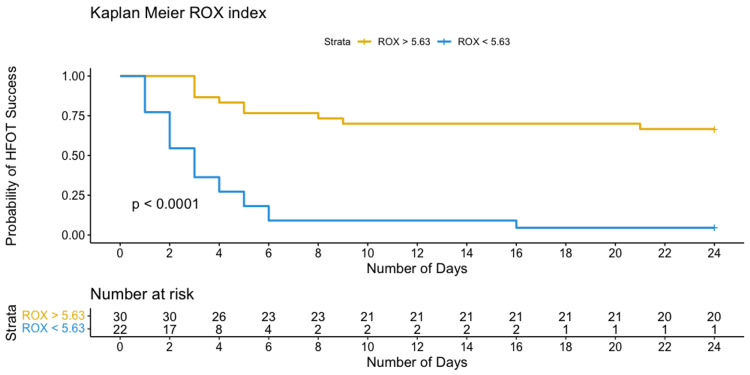
Kaplan-Meier curves of the probability of HFOT failure based on selected thresholds for ROX index. ROX, respiratory rate and oxygenation index; HFOT, high-flow oxygen therapy

**Figure 8:**
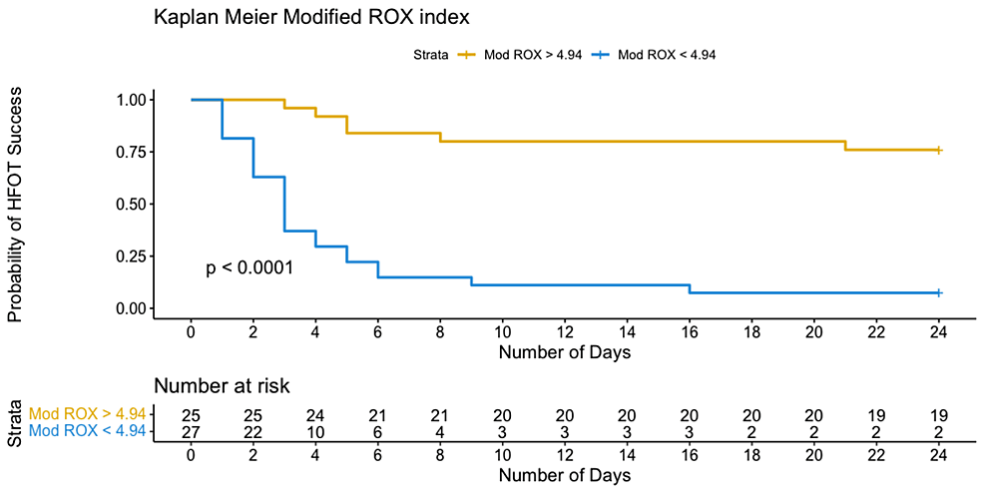
Kaplan-Meier curves of the probability of HFOT failure based on selected thresholds for modified ROX index. ROX, respiratory rate and oxygenation index; HFOT, high-flow oxygen therapy

Patients with values below the upper mentioned thresholds (PaO_2_/FiO_2_ < 123.6, ROX index < 5.63, and modified ROX index < 4.94) required IMV within 10 days after their admission, as depicted by Kaplan-Meier curves in Figures 6-8.

## Discussion

In this retrospective, single-center, observational study, we evaluated the predictive ability of the mean values after the first 24 hours in the ICU of the PaO_2_/FiO_2_ ratio, ROX index, and modified ROX index for the failure of HFOT in patients with COVID-19 AHRF. The statistical reliability of these three models to discriminate between HFOT failure and success, as shown by the multivariate Cox proportional hazard prediction models, are relevant as a screening tool to guide clinical decision-making and to prioritize the available resources.

We calculated the threshold for each tool as a mean, using six measurements from the first 24 hours in the ICU. We considered this approach challenging due to its dynamic assessment of patients’ response to oxygen therapy and ICU care over 24 hours rather than considering only static indexes. Calculating mean values for more than 24 hours would have been less relevant due to the decrease in the statistical power and increase of a type II error.

Our threshold of the PaO_2_/FiO_2_ ratio of 123.6 showed good sensitivity (87%) and specificity (81%) with an area under the receiver operating characteristic curve (AUROC) of 0.85 to predict HFOT failure. Several thresholds have been mentioned in the literature, of which 90 and 62.5 mmHg were statistically correlated with HFOT failure [24].

The ROX index achieved a sensitivity of 68% and a good specificity of 95% (AUROC 0.848) to predict HFOT failure with a threshold of 5.63. Although the ROX index has been implemented for the assessment of acute respiratory failure secondary to pneumonia by Roca et al. [12], recent studies have supported its use in COVID-19 AHRF with noninvasive ventilation. Before the COVID-19 era, the ROX index cutoff point for HFOT success was defined at 4.88 and measured 12 hours after the onset of HFOT.

Several studies have evaluated the feasibility of the ROX index in AHRF: Fink et al. [16] showed that the ROX index accurately predicted the failure of therapy in COVID-19 AHRF at values under 4.88 at 2 hours (AUROC 0.78 and 95% CI 0.67-0.90) and 12 hours (AUROC 0.82 and 95% CI 0.70-0.94). Another retrospective study reported the ROX index threshold at 4 hours of 5.37 (AUROC 0.75, sensitivity 66%, specificity 83%, HR 0.59, and 95% CI 0.41-0.84) [25]. Additionally, Panadero et al. [26] suggested that an ROX index < 4.97 measured 2-6 hours after HFOT initiation could significantly predict an increase in IMV requirements (HR 4.03, 95% CI 1.18-13.7, and *P* = 0.026). A prospective observational study by Ferrer et al. [27] showed a cutoff score of 5.35 for failure at any time up to 24 hours after HFOT initiation (sensitivity 91%, specificity 79%, and HR 0.39).

Another example is a multicenter retrospective study conducted by Chandel et al. [17], which reported an ROX index of 3.67 measured at 12 hours under HFOT to statistically predict therapy failure for values below (AUROC 0.78, sensitivity 84.1%, specificity 49.4%, and 95% CI 0.72-0.84); they also stated that prolonged use of HFOT was not associated with worse clinical outcomes. In addition, a study by Vega et al. [28] showed that the ROX index of 5.99 measured at 12 hours had the best predictor value of HFOT failure and of the risk of endotracheal intubation among values taken at 2, 6, 12, and 24 hours (AUC 0.791, sensitivity 62%, specificity 96%, and 95% CI 0.69-0.89). Recently, Myers et al. investigated retrospectively the ROX index's positive predictive value for the risk of IMV in AHRF and suggested a threshold of 3.85 or less in their cohort [29].

The modified ROX index was suggested by Karim and Esquinas [22] as an alternative to the ROX index. After incorporating the PaO_2_/FiO_2_ ratio into the ROX index formula, and based on the mean of six measurements over 24 hours in our study, we showed that a threshold of 4.94 predicted HFOT failure (AUROC 0.872, sensitivity 81%, and specificity 90%) and had the best performance among all measures. The usefulness of the modified ROX index may be applied in situations where improved sensitivity and specificity performance is needed to identify patients at higher risk of P-SILI and those who would benefit from IMV. In a recent retrospective study, Li et al. [30] examined the same modified ROX index for predicting HFOT outcomes, and their threshold value at 2 hours since therapy initiation at 4.3 had the highest sensitivity (96.1%) and at 7.1 for the highest specificity (100%), concluding that modified ROX perform better than ROX index at predicting HFOT response.

Our study has several limitations. First, the design was a single-center retrospective study in a regional hospital over a short period. Second, the study had a small sample size. Third, for patients who received intravenous dexmedetomidine, the scores we measured may have been influenced by the pharmacodynamics of the drug over the respiratory center (increased comfort). Fourth, the multivariate Cox proportional hazard model for PaO_2_/FiO_2_ did not use a modified SOFA score for RR. Fifth, the time between the disease onset, admission to the ICU, and HFOT administration was heterogeneous, without increasing the selection bias as all patients met the inclusion criteria. Sixth, chest CT imaging findings were quantitatively evaluated by a radiologist for patchy ground glass opacities and consolidation without using a standardized method, which may expose the cohort to interpretation bias. Seventh, the usage of PaO_2_/FiO_­2_ measurements in our variables may limit their application to specific hospital units, such as ICUs and emergency departments. Eighth, the study included patients infected during the Alpha variant wave in Belgium and the vaccines were not yet available.

In particular, during the study period, there was no clear consensus on the benefits of awake prone positioning in patients with AHRF caused by COVID-19. However, it is now evident that this procedure would have influenced the trends of the studied tools.

Finally, our approach offers a new perspective on the assessment of the ROX index and modified ROX index thresholds as dynamic and reliable predictors of HFOT failure. Even if our thresholds are within the range of ROX thresholds quoted, they need external validity.

## Conclusions

The results of this study suggest that early assessment through mean values of the PaO_2_/FiO_2_ ratio, ROX index, and modified ROX index can successfully and accurately identify patients likely to experience HFOT failure under COVID-19 AHRF. However, as these cutoff points remain debated and still insufficiently validated, we advocate using these thresholds with caution in deciding the time of intubation. In our cohort, higher cutoff scores were associated with HFOT success.
